# BACH2-mediated CD28 and CD40LG axes contribute to pathogenesis and progression of T-cell lymphoblastic leukemia

**DOI:** 10.1038/s41419-024-06453-8

**Published:** 2024-01-17

**Authors:** Min Feng, Bailing Zhang, Guilan Li, Yan Yang, Jiangyuan Liu, Ziting Zhang, Bing Zhou, Han Zhang

**Affiliations:** 1 Institute of Medical Biology, Chinese Academy of Medical Sciences and Peking Union Medical College, Kunming, Yunnan 650118 China; 2 State Key Laboratory of Stem Cell and Reproductive Biology, Institute of Zoology, Chinese Academy of Sciences, Beijing, 100101 China; 3 Beijing Institute for Stem Cell and Regenerative Medicine, Institute for Stem Cell and Regeneration, Chinese Academy of Sciences, Beijing, 100101 China; 4 University of Chinese Academy of Sciences, Beijing, 100049 China

**Keywords:** Acute lymphocytic leukaemia, Targeted therapies, Target identification

## Abstract

T-cell acute lymphoblastic leukemia (T-ALL) is an aggressive subtype of ALL characterized by its high heterogeneity and unfavorable clinical features. Despite improved insights in genetic and epigenetic landscapes of T-ALL, the molecular mechanisms that drive malignant T-cell development remain unclear. BTB and CNC homology 2 (BACH2) is a lymphoid-specific transcription repressor recognized as a tumor suppressor in B-cell malignancies, but little is known about its function and regulatory network in T-ALL. Here we found extremely low levels of *BACH2* in T-ALL clinical samples and cell lines compared to normal T cells. Overexpression of BACH2 in T-ALL cells not only induced cell growth retardation but also inhibited cancer progression and infiltration in xenografts. Further RNA sequencing (RNA-seq) analysis revealed significant alterations in regulation of defense and immune responses in T-ALL cells upon BACH2 overexpression. Strikingly, CD28 and CD40LG, two essential stimulatory molecules on T cells, were for the first time identified as novel downstream targets repressed by BACH2 in T-ALL cells. Interestingly, both CD28 and CD40LG were indispensable for T-ALL survival, since largely or completely silencing CD28 and CD40LG led to rapid cell death, whereas partial knockdown of them resulted in cell-cycle arrest and enhanced apoptosis. More importantly, BACH2-mediated CD28 and CD40LG signals contributed to cell migration and dissemination of T-ALL cells to the bone marrow, thus adding a new layer to the BACH2-mediated tumor immunoregulation in T-cell malignancies.

## Introduction

T-cell acute lymphoblastic leukemia (T-ALL) is an aggressive subtype of ALL, accounting for approximately 15% of pediatric and 25% of adult ALL cases [1]. T-ALL is characterized by its high heterogeneity and unfavorable clinical features including high leukocytes, hematopoietic failure, and central nervous system infiltration. Despite the therapeutic advances in T-ALL, patients often suffer from chemoresistance and disease relapse, leading to treatment failure. Severe toxicity caused by intensive multiagent chemotherapy is another great challenge. One of the major obstacles in the management of T-ALL is the ill-defined molecular leukemogenesis, resulting in the lack of specific therapeutic targets. Although several mutations and chromosomal changes have been identified in most cases and the roles of many oncogenes have been studied, for example the activating mutations of notch receptor 1 (*NOTCH1*) [2], the γ-secretase inhibitors (GSIs) targeting NOTCH1 signaling have shown unsatisfactory therapeutic effect on T-ALL [3], suggesting that more studies are urgently needed.

BTB and CNC homology 2 (BACH2) is a lymphoid-specific transcription repressor characterized by its crucial roles in the development and differentiation of lymphocytes. In B cells, BACH2 promotes cell development by repressing myeloid genes in common lymphoid progenitor stage and regulates class-switch recombination and the somatic hypermutation of immunoglobulin genes [4, 5]. Therefore, it is not surprising that aberrant expression of BACH2 is associated with B-cell malignancies. Indeed, we and others have revealed a tumor-suppressor role of BACH2 in B-cell leukemias and non-Hodgkin’s lymphomas (NHLs) [6–10]. The decreased BACH2 levels in B-cell leukemias are primarily attributed to the genetic lesions of its upstream activator paired box 5 [8], whereas one primary mechanism underlying the loss of BACH2 in B-cell NHLs is the frequent deletion of the long arm of chromosome 6, a region where the *BACH2* gene is located [11].

In addition, BACH2 also maintains T cells in a naïve state by suppressing effector memory-related genes [12], and stabilizes regulatory T (Treg)-mediated homeostasis [13]. As such, BACH2 has largely been linked to autoimmune and allergic diseases [14, 15]. However, the function of BACH2 in T-cell malignancies has yet to be determined. Interestingly, we previously revealed the decreased *BACH2* levels in patients with T-ALL compared to those with B-ALL at newly diagnosis (ND), in line with the poorer outcome of T-ALL than B-ALL [10]. Additionally, the lower the *BACH2* levels in T-ALL patients at ND, the longer the duration of minimal residual disease-positive status [10]. Of particular interest, BACH2-mediated downstream regulatory network varies between B-ALL and T-ALL clinical samples [10], highlighting distinct regulatory functions of BACH2 in B- and T-cell malignancies. Considering the above, it is vital to fully depict the roles of BACH2 and its downstream network in T-ALL, thereby uncovering BACH2-mediated mechanisms in T-ALL pathogenesis and progression.

In the present study, we performed in vitro and in vivo experiments, together with clinical data analysis, RNA sequencing (RNA-seq) and cleavage under targets and tagmentation sequencing (CUT&Tag-seq), to investigate the roles of BACH2 and its downstream targets in T-ALL. Remarkably, we found that BACH2, albeit at low levels in T-ALL cells, is closely linked to cell growth, survival, and bone marrow (BM) infiltration. We also revealed for the first time the BACH2-CD28 and BACH2-CD40LG axes in the progression and dissemination of T-ALL, adding a new layer to the landscape of BACH2-mediated tumor immunoregulation in T-cell malignancies.

## Results

### Expression feature of BACH2 in patients with T-ALL and tumor–suppressor-like role of BACH2 in T-ALL cells and xenografts

**Fig. 1:**
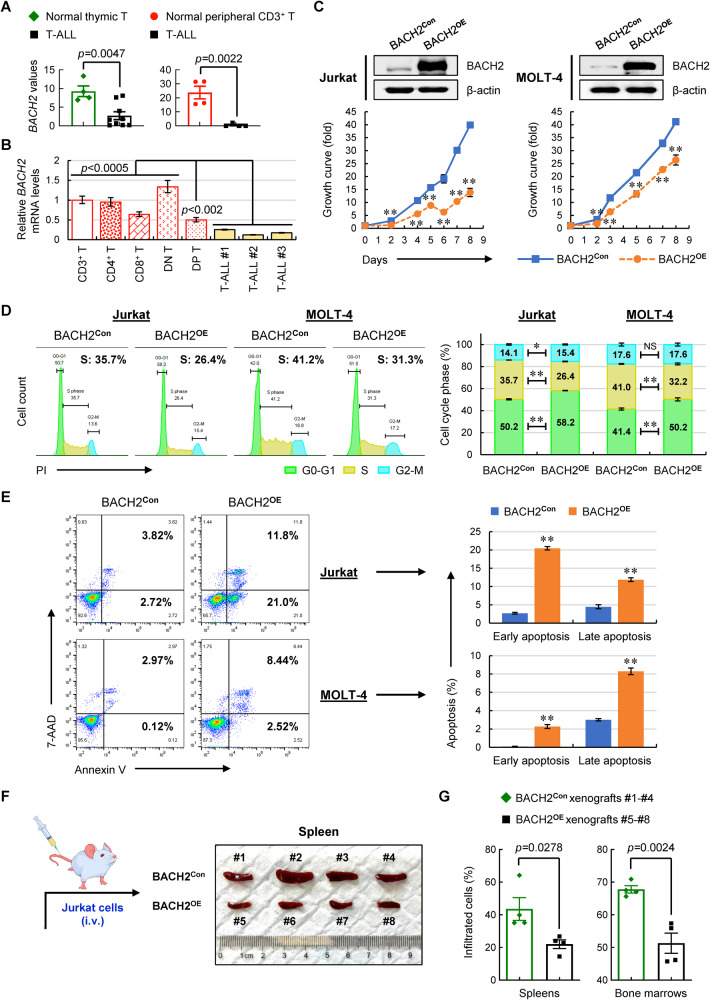
Decreased BACH2 levels in T-ALL patients and tumor-suppressor-like role of BACH2 in T-ALL cells and xenografts. **A** *BACH2* expression values in T-ALL patient samples (*n* = 9) vs. normal thymic T cells (*n* = 4) from GSE63602 (left), as well as in T-ALL patient samples (*n* = 4) vs. normal peripheral CD3^+^ T cells (*n* = 4) from GSE26530 (right). **B** Relative *BACH2* mRNA levels in three T-ALL patient samples (#1-#3) vs. normal peripheral CD3^+^ T cells and different T-cell subsets including DN, SP (CD4^+^ T and CD8^+^ T) and DP T cells. The fold change in expression relative to the *BACH2* levels of CD3^+^ T cells is shown as the mean ± SD. Each of the T-cell subsets is compared to three T-ALL samples, respectively, with the *p* values indicated. **C** The overexpression efficiency of BACH2 (BACH2^**OE**^) in Jurkat (left) and MOLT-4 (right) T-ALL cells were validated by immunoblots with a nonsilencing shRNA plasmid (BACH2^**Con**^) as a negative control. β-actin was used as a loading control. Viable cells were counted in manipulated Jurkat and MOLT-4 cells (lower). **D** Representative cell-cycle distribution of manipulated Jurkat and MOLT-4 cells (left). The % population of cells in each phase is shown as the mean ± SD from three independent experiments (right). **E** Representative cell apoptosis in manipulated Jurkat and MOLT-4 cells staining with Annexin V/7-AAD (*left*). The % population of early and late apoptotic cells in each group is shown as the mean ± SD from three independent experiments (right). NS not significant; *p < 0.05; **p < 0.01 (vs. control group). **F** Manipulated Jurkat cells (2 × 10^6^ cells/mouse) were intravenously (i.v.) injected into NOD/SCID mice via tail vein (*n* = 8). Mice #1 to #4 were injected with BACH2^**Con**^ cells, whereas mice #5 to #8 were injected with BACH2^**OE**^ cells. Xenografts were sacrificed when they presented leukemic phenotypes. Spleens were isolated and photographed against a ruler in centimeters. **G** GFP^+^ cells from spleens (left) and bone marrows (right) in each group were analyzed using flow cytometry. Data are presented as the mean ± SEM.

To determine the clinical relevance of BACH2 in T-ALL, we first analyzed the expression levels of *BACH2* based on publicly available RNA-seq datasets. As shown in Fig. 1A, leukemic cells from patients with T-ALL showed significantly decreased *BACH2* levels compared to normal thymic T cells (GSE63602), and the same is true when we compared T-ALL samples with normal peripheral CD3^+^ T cells (GSE26530). This finding was further validated by an independent cohort of T-ALL patients at ND (*n* = 3) showing that *BACH2* levels were remarkably decreased in leukemic cells compared with normal peripheral CD3^+^ T cells and different T-cell subsets including single-positive (SP), double-negative (DN), and double-positive (DP) T cells (Fig. 1B). Likewise, two human T-ALL cell lines, Jurkat and MOLT-4, also showed dramatically reduced *BACH2* levels compared with normal peripheral CD3^+^ T cells (Supplementary Fig. S1). These data provided a strong indication that *BACH2* is downregulated in T-ALL cells.

Given that T-ALL cells have low basal levels of BACH2, we generated stable BACH2-overexpressing (BACH2^**OE**^) cells in Jurkat and MOLT-4 (Fig. 1C). Compared with control cells (BACH2^**Con**^), BACH2^**OE**^ cells showed reduced growth rates (Fig. 1C). Cell-cycle distribution also displayed a cell-cycle arrest in G0-G1 phase and an approximately 9% of decrease in S-phase population upon BACH2 overexpression (Fig. 1D). Further cell survival analysis revealed a remarkable apoptosis in BACH2^**OE**^ T-ALL cells compared to control cells, especially in Jurkat cells (Fig. 1E), coinciding with the lower growth rate in Jurkat than that in MOLT-4 after BACH2 overexpression. Moreover, the phenotypes induced by BACH2 overexpression in T-ALL cells can be reversed by reduction in BACH2 levels (Supplementary Fig. S2A–C), further supporting a tumor-suppressor-like role of BACH2 in T-ALL cells.

To confirm whether BACH2 levels affect organ infiltration by leukemic T cells, we intravenously (i.v.) transplanted the manipulated Jurkat cells into NOD/SCID mice. As shown in Fig. 1F, BACH2^**OE**^ T-ALL xenograft mice exhibited smaller spleens than those in control group. Further analysis showed lower percentages of the infiltrated T-ALL cells in the spleens and BMs from the BACH2^**OE**^ xenografts than those from control group (Fig. 1G), indicating decreased leukemia burden. This finding, together with the in vitro phenotypes, supported the notion that overexpression of BACH2 in T-ALL cells inhibits cancer progression and T-ALL infiltration.

### Increased BACH2 levels induce significant alterations in regulation of defense and immune responses in T-ALL cells

**Fig. 2:**
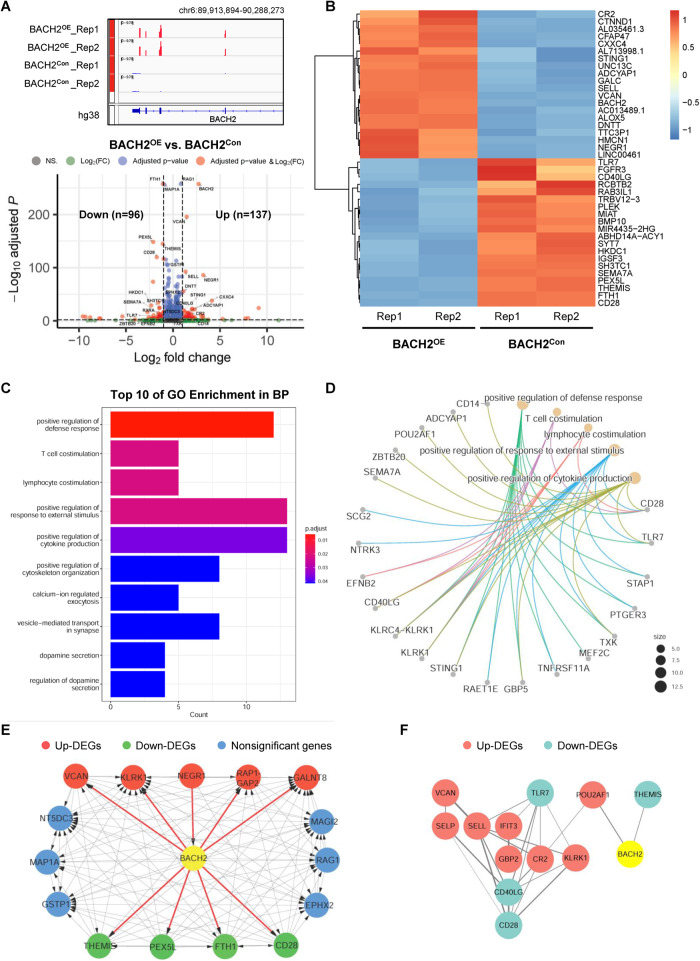
RNA-seq analysis of manipulated T-ALL cells. **A** Visualization of *BACH2* transcriptional levels (red peaks) with IGV from two replicates in each group. GRCh38 genome was used as a human reference genome (*upper*). Volcano plot was generated with 137 Up-DEGs and 96 Down-DEGs in BACH2^**OE**^ Jurkat cells vs. control cells (*lower*). The Log_2_(FC), adjusted *p* values were calculated using DESeq2 default settings, and significant genes were considered the ones with adjusted *p* values < 0.05. **B** Heatmap of the top 20 Up- and Down-DEGs from two replicates in each group. The level of gene expression is indicated by the color intensity, with red representing high expression and blue representing low expression. **C** Top 10 of GO terms enriched in BP category based on the enrichment analysis of the obtained DEGs. **D** DEGs that were enriched in the top 5 GO-BP terms. **E** Top 15 hub genes in BACH2-involving gene regulatory network using weighted gene co-expression network analysis. The black arrows pointed to the downstream target genes and the lines indicated the gene-gene interactions. Red, green, and blue represent the Up-DEGs (*n* = 5), Down-DEGs (*n* = 4), and nonsignificant genes (*n* = 6), respectively. **F** Top 12 hub nodes in BACH2-involving PPI network, with red representing the Up-DEGs (*n* = 8) and blue representing the Down-DEGs (*n* = 4).

Despite the antitumor-like features of BACH2 in T-ALL cells, the mechanisms underlying BACH2-mediated signaling in T-ALL cells remain unclear. To delineate the regulatory network downstream of BACH2 in T-ALL cells, RNA-seq was utilized to capture the transcriptome changes mediated by BACH2 in Jurkat cells due to their remarkable phenotypes. Total mRNAs from BACH2^**OE**^ Jurkat cells were compared to the control group, and 137 differentially expressed genes (DEGs) were upregulated while 96 DEGs were downregulated (Fig. 2A). Principal component analysis (PCA) revealed distinct alterations in transcriptional signatures of BACH2^**OE**^ cells versus (vs.) control cells (Supplementary Fig. S3A). The resulting heatmap also supported two hierarchical clusters on the similar gene expression profiles from two replicates in each group (Fig. 2B). To test the robustness of DEGs analysis, the top upregulated DEGs (Up-DEGs) and downregulated DEGs (Down-DEGs) were validated in the manipulated MOLT-4 cells, which showed consistent expression patterns with RNA-seq (Supplementary Fig. S3B). By contrast, in BACH2-lower T-ALL patient samples, the expression patterns of many DEGs were inverse to those in transcriptome analysis (Supplementary Fig. S3C).

Further Gene Ontology (GO) enrichment analysis of DEGs in biological process (BP) revealed “positive regulation of defense response” as the top-ranked term (Fig. 2C), followed by “positive regulation of response to external stimulus” and “positive regulation of cytokine production”, suggesting that BACH2 is very likely to play a positive regulatory role in immune defense. The second and third enriched GO terms were “T-cell costimulation” and “lymphocyte costimulation”, highlighting the crucial roles of BACH2 in regulating lymphocytes. Indeed, the DEGs that were enriched in the top 5 GO terms included *CD28* and *CD40LG* that are essential for T-cell activation, proliferation, and survival, as well as *TLR7*, *GBP5*, *RAET1E*, *STING1*, *KLRK1*, *KLRC4-KLRK1* and *ZBTB20* that are major regulators in activation of innate immunity (Fig. 2D).

To further identify the gene modules that are highly correlated with BACH2 in T-ALL cells, a weighted gene co-expression network was constructed (Supplementary Fig. S3D), in which the black arrows pointed to the downstream target genes and the lines indicated the gene-gene interactions. Among these modules, the top 15 hub genes included 5 Up-DEGs, 4 Down-DEGs, and 6 nonsignificant genes (Fig. 2E), of which, *CD28*, *THEMIS*, *KLRK1* and *VCAN* were also identified as hub nodes in the top 12 of BACH2-involving protein-protein interaction (PPI) network (Fig. 2F).

### Identification of the downstream transcriptional targets of BACH2 in T-ALL cells

**Fig. 3:**
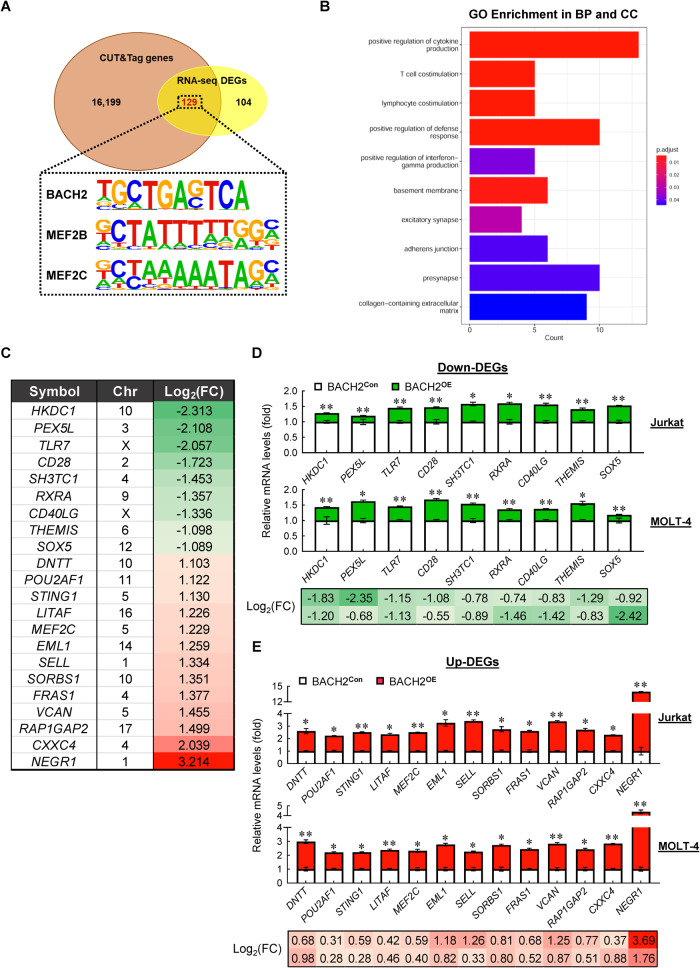
Identification of the downstream transcriptional targets of BACH2 in T-ALL cells. **A** Venn diagram showing the overlap of annotated peaks containing promoter region identified from CUT&Tag-seq for BACH2 and the DEGs from RNA-seq. **B** GO enrichment analysis of the overlapping genes (*n* = 129) including the top 5 terms in BP category and the top 5 terms in cellular component (CC) category. **C** Log_2_(FC) of the selected 22 DEGs from RNA-seq. Each value from the RNA-seq in BACH2^**OE**^ Jurkat cells was relative to the control cells. Chr, chromosome. Relative mRNA levels of Down-DEGs (*n* = 9) **D** and Up-DEGs (*n* = 13) **E** in manipulated Jurkat and MOLT-4 cells. Each value was normalized to *ACTB* and is presented as the mean ± SD from three independent experiments. *p < 0.05; **p < 0.01 (vs. control group). Log_2_(FC) of each gene in BACH2^**OE**^ cells relative to the control cells is indicated under the bar chart, with the upper row representing Jurkat and the lower row representing MOLT-4.

We next wondered which DEGs are the downstream targets regulated by BACH2 in T-ALL cells. To this end, we performed CUT&Tag-seq in Jurkat cells using the antibodies specific for human BACH2. The integration analysis of RNA-seq and CUT&Tag-seq revealed 162 overlapping genes (Supplementary Fig. S4A). Of this gene set, 129 genes with peaks containing promoter region were further extracted (Fig. 3A), which were preferentially enriched in the “positive regulation of cytokine production”, “T-cell costimulation”, “lymphocyte costimulation”, “positive regulation of defense response”, and “positive regulation of interferon-gamma production” (Fig. 3B). Intriguingly, these genes harbor not only BACH2-binding motif (5’-TGCTGAG/CTCA-3’) but also the motifs of MEF2B and MEF2C, two members of the myocyte-enhancer factor 2 (MEF2) family [16] (Fig. 3A). Notably, MEF2B plays an essential role in early germinal center (GC) formation by directly activating *BCL6* transcription [17], whereas MEF2C is required for the proliferation and survival of B cells by activating *BCL2L1* transcription [18]. Given the fact that BACH2 is involved in GC B-cell differentiation [19], these 129 genes may also play crucial roles in the development of B cells.

Among these 129 genes, we further selected 22 genes for validation. They included 9 Down-DEGs and 13 Up-DEGs (Fig. 3C), which were high-ranking DEGs with the adjusted *p* value less than 10^4^ and most of them were identified as potential BACH2-interacting genes (Fig. 2E, F). Consistent with the Log_2_ fold change (FC) from RNA-seq (Fig. 3C), we confirmed the downregulation (Fig. 3D) and upregulation (Fig. 3E) of these genes in manipulated Jurkat and MOLT-4 cells vs. control cells. Most of the genes also exhibited inverse expression patterns in BACH2-low T-ALL patient samples compared with normal peripheral CD3^+^ T cells; however, not all genes reached statistical significance (Supplementary Fig. S4B, C), suggesting that other events or mechanisms are concurrently involved in the regulation of these genes.

### CD28 and CD40LG are novel downstream targets repressed by BACH2 in T-ALL cells

**Fig. 4:**
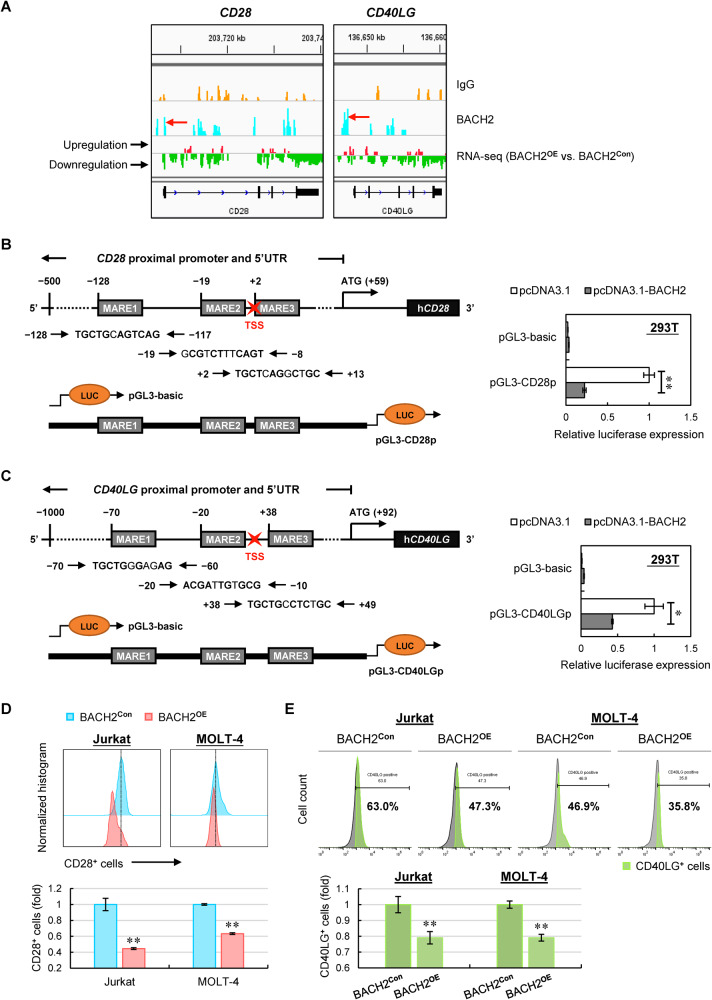
CD28 and CD40LG are novel downstream targets repressed by BACH2 in T-ALL cells. **A** Visualization of BACH2 peaks at *CD28* (*left*) and *CD40LG* (right) promoters with orange reads representing antibodies against control immunoglobulin G (IgG) and blue reads representing antibodies against human BACH2. Transcriptional levels of the *CD28* and *CD40LG* genes from RNA-seq are indicated with red reads representing upregulation while green reads representing downregulation. GRCh38 genome was used as a human reference genome. Truncated promoter construct containing three putative BACH2-binding sites (MARE1-3) of the *CD28* gene (**B**) or the *CD40LG* gene (**C**) is indicated (left). 293T cells were transfected with truncated promoter plasmids, BACH2 expression plasmids, or control plasmids (pcDNA3.1). An empty pGL3-basic plasmid was used as a negative control. The *Renilla* luciferase reporter pRL-SV40 was used as an internal control for normalization. Luciferase activity was measured 48 h after transfection. Data are presented as the relative luciferase activity compared with the cells transfected with control plasmids (right). Data are shown as the mean ± SD from three independent experiments. **D** Representative expression levels of CD28 in manipulated Jurkat and MOLT-4 cells using flow cytometry (upper). The relative intensity of CD28 positive (CD28^+^) cells were normalized to control cells and shown as the mean ± SD from three independent experiments (lower). **E** Representative expression levels of CD40LG in manipulated Jurkat and MOLT-4 cells using flow cytometry (upper). The % population of CD40LG positive (CD40LG^+^) cells were normalized to control cells and shown as the mean ± SD from three independent experiments (lower). *p < 0.05; **p < 0.01 (vs. control group).

Since BACH2 is a transcriptional repressor, the Down-DEGs upon BACH2 overexpression aroused our great interest. In this context, *CD28* and *CD40LG* stood out from the validated 9 Down-DEGs containing the BACH2-binding motif (Fig. 3C). Firstly, the CUT&Tag-seq analysis showed significant BACH2-enriched peaks within the proximal promoter regions of *CD28* and *CD40LG* genes, which were significantly downregulated in BACH2^**OE**^ cells vs. control cells from RNA-seq (Fig. 4A). Secondly, CD28 and CD40LG are two essential stimulatory molecules expressed on the surface of T cells [20, 21]; aberrant overexpression or activation of them contribute to many types of T-cell malignancies including adult T-cell leukemia/lymphoma (ATLL) and T-cell lymphomas [22–28]. Considering the above, we reasoned that *CD28* and *CD40LG* are likely the downstream targets repressed by BACH2 in T-ALL cells.

To test our hypothesis, luciferase reporter was constructed by fusing the enriched peak region of *CD28* or *CD40LG* promoter with the luciferase gene (Fig. 4B, C). Ectopic expression of BACH2 in 293 T cells largely decreased the luciferase activity of *CD28* promoter (Fig. 4B) and *CD40LG* promoter (Fig. 4C), respectively. Further flow cytometry analysis confirmed the reduced protein levels of CD28 (Fig. 4D) and CD40LG (Fig. 4E) in T-ALL cells upon BACH2 overexpression. These findings, along with the CUT&Tag-seq results, strongly supported that BACH2 is a transcriptional repressor of *CD28* and *CD40LG* by binding to their promoters in T-ALL cells.

### Silencing CD28 or CD40LG in T-ALL cells inhibits cell growth by inducing cell-cycle arrest and remarkable apoptosis

**Fig. 5:**
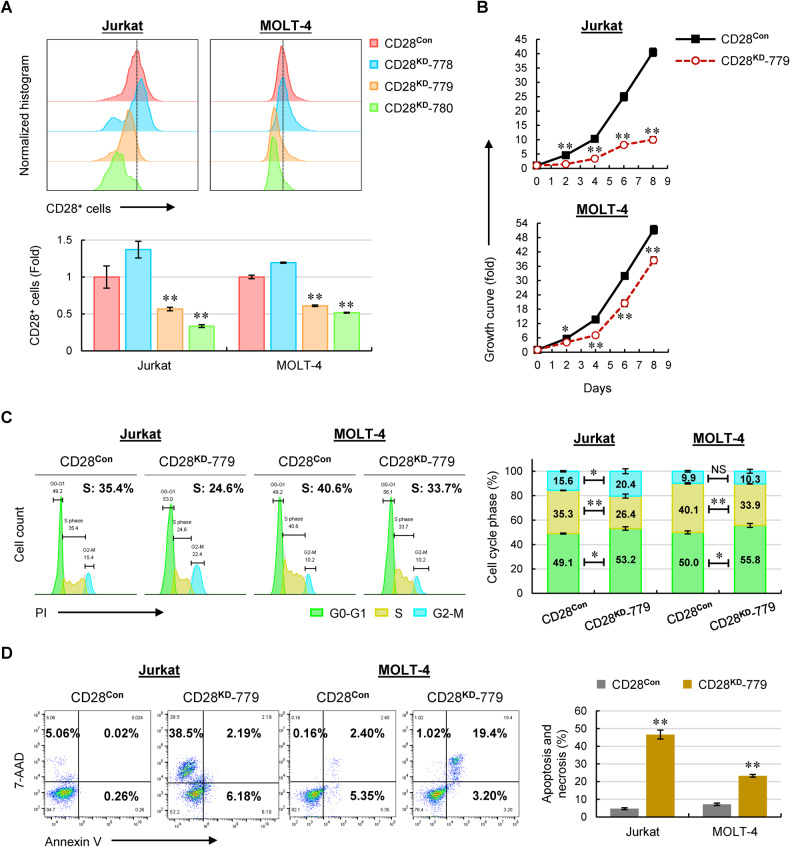
Knockdown of CD28 in T-ALL cells inhibits cell growth by inducing cell-cycle arrest and remarkable apoptosis. **A** Representative expression levels of CD28 in manipulated Jurkat and MOLT-4 cells using flow cytometry (upper). The relative intensity of CD28^+^ cells were normalized to control cells and shown as the mean ± SD from three independent experiments (lower). **B** Viable cells were counted in manipulated Jurkat and MOLT-4 cells. **C** Representative cell-cycle distribution of manipulated Jurkat and MOLT-4 cells (left). The % population of cells in each phase is shown as the mean ± SD from three independent experiments (right). **D** Representative cell apoptosis and necrosis in manipulated Jurkat and MOLT-4 cells staining with Annexin V/7-AAD (left). The % population of apoptotic and necrotic cells in each group is shown as the mean ± SD from three independent experiments (right). NS not significant; *p < 0.05; **p < 0.01 (vs. control group).

Next, we questioned whether CD28 and CD40LG are functionally involved in the progression of T-ALL. To this end, we silenced CD28 (CD28^**KD**^) in two human T-ALL cell lines. The knockdown efficiency of CD28 in T-ALL cells was evaluated by flow cytometry which showed better knockdown efficiency of CD28^**KD**^-780 than CD28^**KD**^-779, whereas no knockdown effect was observed in the CD28^**KD**^-778 clone (Fig. 5A). Surprisingly, an overwhelming apoptosis or necrosis was triggered in CD28^**KD**^-780 cells (Supplementary Fig. S5A), resulting in rapid cell death. This finding indicated that CD28 is an indispensable molecule in T-ALL cells and there is a dose-dependent effect of CD28 expression on cell survival: the more the degree of CD28 knockdown the higher the percentages of apoptosis and necrosis. In this case, CD28^**KD**^-779 cells were used to perform the subsequent functional experiments. Compared with control cells (CD28^**Con**^), CD28 silencing significantly inhibited cell growth, especially in Jurkat cells (Fig. 5B), indicating a potential leukemia-promoting role of CD28 in T-ALL cells. Further cell-cycle analysis revealed a G0-G1 arrest and a significant decrease in S phase upon CD28 silencing (Fig. 5C). Correspondingly, cell survival analysis displayed a remarkable apoptosis or necrosis in CD28^**KD**^-779 T-ALL cells compared to control cells (Fig. 5D).

**Fig. 6:**
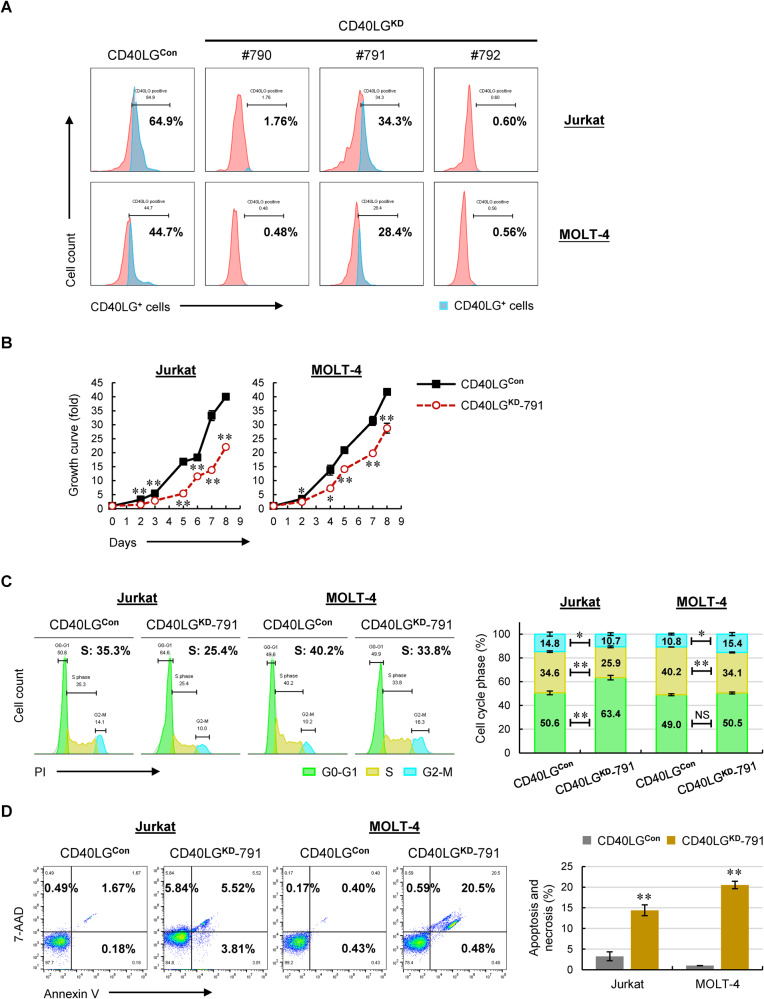
Knockdown of CD40LG in T-ALL cells inhibits cell growth by inducing cell-cycle arrest and apoptosis. **A** Representative expression levels of CD40LG in manipulated Jurkat and MOLT-4 cells using flow cytometry. **B** Viable cells were counted in manipulated Jurkat and MOLT-4 cells. **C** Representative cell-cycle distribution of manipulated Jurkat and MOLT-4 cells (left). The % population of cells in each phase is shown as the mean ± SD from three independent experiments (right). **D** Representative cell apoptosis and necrosis in manipulated Jurkat and MOLT-4 cells staining with Annexin V/7-AAD (left). The % population of apoptotic and necrotic cells in each group is shown as the mean ± SD from three independent experiments (right). NS not significant; *p < 0.05; **p < 0.01 (vs. control group).

Next, we silenced CD40LG (CD40LG^**KD**^) in T-ALL cell lines. As shown in Fig. 6A, CD40LG proteins were nearly completely silenced in CD40LG^**KD**^-790 and CD40LG^**KD**^-792 clones, whereas CD40LG^**KD**^-791 cells exhibited a decrease of CD40LG expression by approximately half. Like CD28^**KD**^-780 cells, both CD40LG^**KD**^-790 and CD40LG^**KD**^-792 cells underwent an overwhelming apoptosis followed by ultimate cell death (Supplementary Fig. S5B), demonstrating an essential role of CD40LG in sustaining T-ALL survival. As such, we used CD40LG^**KD**^-791 cells to perform the functional experiments. Similarly, silencing CD40LG resulted in a reduced cell growth (Fig. 6B), which was mainly attributed to a significant decrease in S phase (Fig. 6C) and a remarkable apoptosis (Fig. 6D) compared with control cells. To sum up, largely or completely silencing CD28 and CD40LG led to rapid cell death, whereas partial knockdown of CD28 or CD40LG resulted in both cell-cycle arrest and decreased cell survival, thus inhibiting the progression of T-ALL.

### Activation of BACH2-mediated CD28 and/or CD40LG signals promotes cell migration and dissemination of T-ALL cells to the BM

Leukemic cells quit the thymus to home to the BM early after the disease spreading, suggesting that the BM is a warm nest for T-ALL [3, 29]. BM-resident T-ALL cells are sheltered from immune attack and chemotherapy within the BM niches, in which bone marrow stromal cells (BMSCs) protect leukemic cells by secreting multiple pro-survival cytokines and chemokines [3]. Since BACH2^**OE**^ xenografts showed low leukemia burden in the BM (Fig. 1G), we then wondered whether these events are mediated by reduced CD28 and/or CD40LG levels in T-ALL cells upon BACH2 overexpression.

**Fig. 7:**
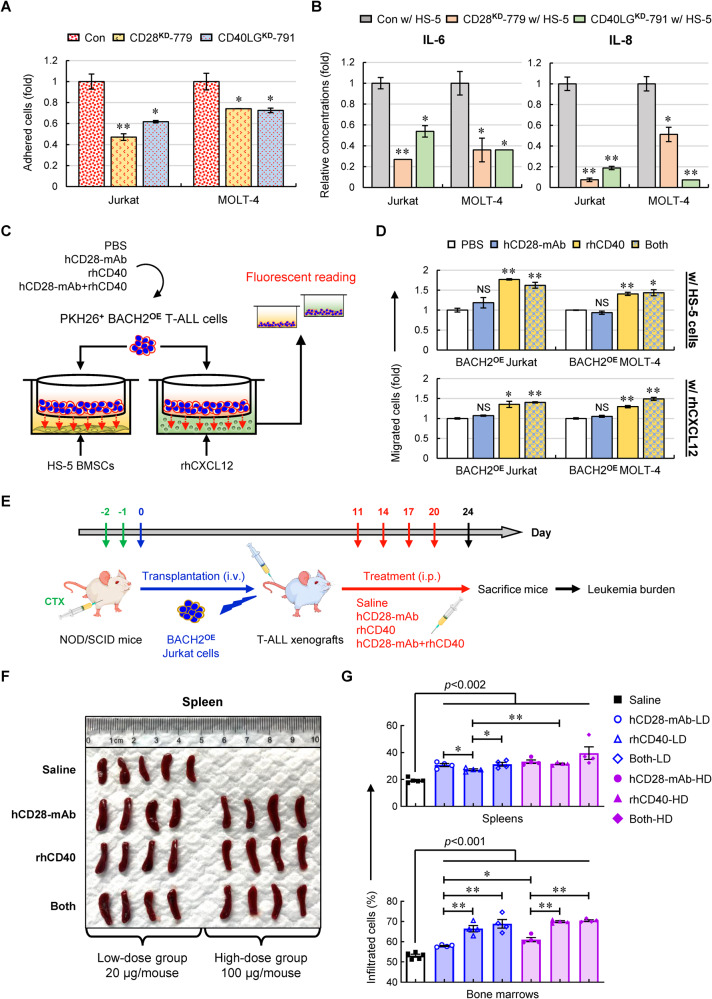
BACH2-mediated CD28 and CD40LG axes contribute to dissemination of T-ALL cells to the BM. **A** Cells were stained with PKH26 prior to seeding onto the pre-established monolayer of HS-5 BMSCs. PKH26 dye intensity in manipulated Jurkat and MOLT-4 cells were normalized to control cells. Data are shown as the mean ± SD from three independent experiments. **B** ELISA analyses of IL-6 and IL-8 using conditioned media upon coculturing CD28^**KD**^ or CD40LG^**KD**^ T-ALL cells with HS-5 cells. The colorimetric values were normalized to the control group. **C** Experimental design for transwell migration assays. Cells were pre-stained with PKH26 followed by the treatment with PBS (control), hCD28-mAb (0.5 μg/mL), rhCD40 (0.5 μg/mL) or both (hCD28-mAb+rhCD40). PKH26 dye intensity of migrated cells in the lower chamber were measured. **D** PKH26 dye intensity of migrated cells was normalized to the control group and shown as the mean ± SD from three independent experiments. **E** Experimental design for testing the effects of activating CD28 and/or CD40LG signals on T-ALL xenografts. **F** Spleens were isolated and photographed against a ruler in centimeters. **G** GFP^+^ cells from spleens (*upper*) and bone marrows (*lower*) in each group were analyzed using flow cytometry. Data are presented as the mean ± SEM. NS not significant; *p < 0.05; **p < 0.01 (vs. control group).

To test our hypothesis, we first used an in vitro coculture model of leukemic cells and BMSCs to investigate the effect of CD28 or CD40LG on cell adhesion and cytokine secretion. Compared with control cells, silencing CD28 or CD40LG in T-ALL cells resulted in a significantly decreased cell adhesion to HS-5 BMSCs (Fig. 7A). Further analysis of the conditioned media exhibited a marked reduction in interleukin-6 (IL-6) and IL-8 secretion when coculturing CD28^**KD**^ or CD40LG^**KD**^ T-ALL cells with HS-5 cells (Fig. 7B), demonstrating alterations in leukemia-infiltrated stromal microenvironment.

Next, we asked whether activation of CD28- and/or CD40LG-mediated signaling reverses BACH2^**OE**^-induced low leukemia burden. To answer this question, we first performed transwell assays, since cell migration is a pivotal process required for T-ALL trafficking to the BM [3]. We mimicked the BM microenvironment using the culture media containing the pre-established monolayer of HS-5 BMSCs or C-X-C motif chemokine ligand 12 (CXCL12)-enriched complete media in the lower well; meanwhile, we activated CD28- and/or CD40LG-signaling of BACH2^**OE**^ T-ALL cells with anti-human CD28 activating monoclonal antibodies (hCD28-mAb) and/or recombinant human CD40 proteins (rhCD40) in the upper chamber (Fig. 7C). Using this in vitro system, we observed an approximately 50% increase in migration when stimulating CD40LG or CD28 + CD40LG signals of BACH2^**OE**^ T-ALL cells vs. PBS-treated control group in both enriched settings, whereas no significant increase in migration was observed when stimulating CD28 signaling alone (Fig. 7D). This finding indicated that CD40LG-mediated signaling plays a major role in T-ALL migration to the BM in contrast to CD28 signaling.

To extend our findings to in vivo models, we treated BACH2^**OE**^ T-ALL xenografts with different doses of hCD28-mAb, rhCD40, or both (hCD28-mAb+rhCD40) for a total of 4 injections (Fig. 7E). Compared with the saline-treated group (control), mice treated with low-dose (LD) and high-dose (HD) hCD28-mAb, rhCD40 or both displayed enlarged spleens (Fig. 7F); the infiltrated T-ALL cells in the spleens and BMs were also dramatically increased upon activation of CD28- and/or CD40LG-signaling (Fig. 7G). Particularly, CD40LG-stimulating T-ALL xenografts exhibited remarkably increased BM infiltration in both LD- and HD-groups compared to CD28-stimulating xenografts, consistent with what we found in transwell assays (Fig. 7D). This result highlighted that CD40/CD40LG signaling is a key regulator of T-ALL infiltration to the BM, whereas BACH2-mediated CD28 axis mainly contributes to the progression of T-ALL in spleens.

## Discussion

During the past decade, significant progress has been made in exploring the genetic and epigenetic landscapes of T-ALL [30–32]. However, translating complex molecular events into clinic is faced with great challenges, as the mechanisms that drive malignant T-cell development remain unclear [3]. Thus, there is an urgent need to discover novel signaling pathways, transcription factors or cell surface markers involving in the progression of T-ALL.

Although BACH2 is well known for its role in regulation of B-cell development and differentiation, BACH2 has increasingly been revealed to orchestrate T-cell differentiation and function [15]. For example, BACH2 is required for efficient formation of Treg cells and is critical for the maintenance of immune homeostasis [13]. BACH2 also actively restrains T-cell activation at steady-state and constrains their functional activity [15]. Despite this, little is known about the roles of BACH2 in T-cell malignancies. In the current study, we found strongly downregulated levels of *BACH2* in T-ALL clinical samples and cell lines compared to normal T cells. Further in vitro and in vivo experiments confirmed a tumor-suppressor-like role of BACH2 in T-ALL progression and organ infiltration. Nonetheless, the factors that regulate the expression of BACH2 in normal and malignant T cells remain poorly defined. For example, we found relatively high *BACH2* levels in DN T cells but low *BACH2* levels in DP T cells compared to other normal peripheral T-cell subsets. This finding seems to be in concordance with the physiological roles of BACH2 [12, 13], since peripheral DN T cells are essential for maintaining immune homeostasis under healthy conditions and contribute to immune surveillance and defense [33–35], whereas DP T cells in the periphery exhibit memory-like features [36, 37]. As such, further efforts are needed to delineate the molecular mechanisms that govern BACH2 transcription or post-transcriptional regulation in normal and malignant T cells, thus elucidating the biological function of BACH2 in scenarios of both health and disease.

Due to the vital roles of BACH2 in the development and differentiation of lymphocytes, BACH2 itself is not an ideal therapeutic target. We thus attempted to explore the downstream pathways or effectors regulated by BACH2 in T-ALL cells. Intriguingly, RNA-seq analysis of BACH2^**OE**^ cells revealed “positive regulation of defense response” as the top-ranked GO term, suggesting a positive regulatory effect of BACH2 on immune defense in T cells. In this context, the low expression of BACH2 in T cells may facilitate leukemogenesis and progression by inhibiting immune defense, thereby creating an immunosuppressive microenvironment. In addition, the decreased BACH2 levels in T-ALL cells also promote cancer development by de-repressing other key effectors. CD28 and CD40LG are stimulatory molecules that are essential for T-cell activation and proliferation [20, 21]. The expression levels of CD28 and CD40LG are tightly regulated in normal T cells, as abnormal activation or overexpression of them lead to many types of T-cell malignancies including ATLL and T-cell lymphomas [22–28]. Strikingly, we discovered that CD28 and CD40LG are the downstream targets repressed by BACH2, and both play indispensable roles in sustaining T-ALL cell growth and survival. Our reverse experiments with leukemia xenografts treated with hCD28-mAb and/or rhCD40 further provided strong evidence for this. These data outlined the fact that the reduced expression of BACH2 in malignant T cells confers oncogenic properties at least partly by de-repressing CD28 and CD40LG, thus promoting T-ALL proliferation and progression. In addition, we found an interesting role of CD40/CD40LG signaling in T-ALL infiltration to the BM. This finding is supported by previous works showing that CD40/CD40LG activation not only promotes adhesion of multiple myeloma cells to BMSCs by upregulating IL-6 and vascular endothelial growth factor secretion [38, 39], but also contributes to cancer cell migration and metastasis [40–42]. In this regard, further studies are warranted to elucidate the mechanisms by which CD40/C40LG axis drives BM infiltration in T-ALL.

Despite the leukemia-promoting roles of CD28 and CD40LG in T-ALL cells, we failed to find statistical significance of *CD28* or *CD40LG* overexpression in T-ALL samples vs. normal T cells in public datasets due to the large individual differences (data not shown). This finding was further verified in two T-ALL samples, of which, one exhibited statistically upregulated mRNA levels of *CD28* and *CD40LG* vs. normal T cells while the other did not (Supplementary Fig. S4B). This discovery suggested that in addition to BACH2, other factors are involved in the transcriptional regulation of *CD28* and *CD40LG* in T-ALL cells. On the other hand, the expression of CD28 or CD40LG is likely to be post-transcriptionally regulated in T-ALL cells, as both were identified as hub nodes in BACH2-involving PPI network (Fig. 2F). Thus, it will be of tremendous interest to investigate the protein levels of CD28 and CD40LG in T-ALL samples as well as their effects on T-ALL prognosis.

Apart from the roles of BACH2-CD28 and BACH2-CD40LG axes in T-ALL cells, we also found aberrant IL-6 and IL-8 secretion upon silencing CD28- or CD40LG in T-ALL cells. Of note, increased IL-6 and IL-8 levels are associated with poor response of cancer cells to GSIs [43]. In T-ALL, NOTCH1 signaling promotes IL-6 secretion by BM stromal components [44]. NOTCH1-dysregulated T-ALL cells also contribute to inducing a small group of myeloid cells via an IL-6-dependent pathway [45]. These myeloid-derived cells not only provide signals to directly support T-ALL survival but also facilitate disease progression [45, 46]. These discoveries provide convincing evidence that the alterations of cytokines in leukemia-infiltrated niches are crucial exogenous signals required for T-ALL cell survival and progression. Nevertheless, IL-6 and IL-8 are not the only contributing factors in T-ALL-infiltrated BM microenvironment, as we also revealed remarkable roles of CXCL12 in cell migration when activating CD40LG or CD28 + CD40LG signals of T-ALL cells. This finding indicated that other chemokines or growth factors, independent or in cooperation with BACH2-CD28/CD40LG axes, are required for BM infiltration by leukemic T cells.

In conclusion, we revealed a tumor-suppressor-like role of BACH2 in T-ALL cells and xenografts. Our discovery of BACH2-CD28 and BACH2-CD40LG axes brings a better understanding of the BACH2-mediated tumor immunoregulation during the progression of T-ALL. Blockade of CD28 or CD40LG, including but not limited to genetic or pharmacologic means, mAbs and chimeric antigen receptor-based therapy, will suppress tumor growth and BM infiltration, thereby offering a novel therapeutic approach for the treatment of T-ALL.

## Methods and materials

### Clinical sample processing and analyses

Diagnostic BM samples were obtained from patients with T-ALL (*n* = 3) at ND approved by the Institutional Review Board Committee with informed consent in accordance with the Declaration of Helsinki [10]. Mononuclear cells from BM samples were isolated as previously described [10]. CD3^+^ and T-cell subsets including DN (CD4^−^CD8^−^CD56^−^TCRα/β^+^), DP (CD4^+^CD8^+^), and SP (CD4^+^CD8^−^ or CD4^−^CD8^+^) from normal human peripheral blood mononuclear cells (Cellverse Bioscience Technology Co., Ltd., Shanghai, China) were enriched using MACS separation system (Miltenyi Biotec, Bergisch Gladbach, Germany) according to the manufacturer’s instructions.

Two publicly available RNA-seq datasets (GSE63602 and GSE26530) were utilized for the comparison of *BACH2* levels between T-ALL patient samples at diagnosis and normal T cells. Briefly, the transcripts per million (TPM) normalized expression values of *BACH2* from GEO2R were used for analysis of GSE63602 [47], and the TPM normalized expression values of *BACH2* from the supplementary file (GSE26530_DGE_Sense_GeneExpression_TPM. txt.gz) were used for analysis of GSE26530 [48].

### Cell lines and culture

Human T-ALL cell line Jurkat was purchased and authenticated using short tandem repeat (STR) profiling at BNCC (Beijing, China); human T-ALL cell line MOLT-4 was purchased and authenticated by STR profiling at Cobioer Biosciences (Nanjing, China). Human bone marrow stromal cell line HS-5 was purchased from BNCC (Beijing, China) and authenticated by STR profiling at CinoAsia Institute (Shanghai, China). All cell lines were tested to be free of mycoplasma contamination. Cells were cultured in RPMI1640 or DMEM medium supplemented with 10% fetal bovine serum and 100 U.I./mL penicillin-streptomycin. Cells were maintained under 5% CO_2_ at 37 °C.

### Lentiviral infection and generation of stable cell lines

The overexpressed lentivirus and the shRNA-mediated lentivirus specific for human BACH2 as well as their corresponding control lentiviruses were generated as previously described [10]. Three shRNA-mediated lentiviruses specific for human CD28 (#778, #779 and #780), three shRNA-mediated lentiviruses specific for human CD40LG (#790, #791 and #792), and their corresponding control lentiviruses were purchased from Genechem (Shanghai, China). T-ALL cells were infected with lentiviruses according to the manufacturer’s instructions. The lentiviral-transduced cells were further selected with puromycin (2 μg/mL) and/or blasticidin (5 μg/mL) for 7–14 days, followed by validation of immunoblots or flow cytometry.

### RNA isolation and quantitative real-time PCR (qRT-PCR)

RNA was isolated as previously described [49], followed by qRT-PCR using a One Step SYBR PrimeScript PLUS RT-PCR kit (Takara, Kusatsu, Japan). Each sample was performed in triplicate, and the relative expression of each gene was normalized to the *ACTB* gene by the method of 2^−ΔΔCt^. The involved primers are shown in Supplementary Table S1, and primers for *BACH2* and *ACTB* were provided as described before [9, 50].

### Protein detection by immunoblots and flow cytometry

Harvested cells were lysed to perform immunoblotting assay as previously described [49]. Immunoblots were further subjected to semi-quantitative analysis using an ImageJ software. The relative expression of target proteins was normalized to β-actin. The protein levels of CD28 and CD40LG were detected by flow cytometry on a CytoFLEX flow cytometer (Beckman Coulter).

The following antibodies were used for immunoblots or flow cytometry: anti-BACH2 (#80775), anti-β-actin (#3700), anti-mouse (#7076) and anti-rabbit (#7074) secondary antibodies were purchased from Cell Signaling (Danvers, MA, USA); anti-CD28 (#560683) and anti-CD40LG (#560955) were purchased from BD Biosciences (San Jose, CA, USA). Full and uncropped immunoblots are presented in Supplemental File.

### Cell-cycle and apoptosis assays

Cell cycle and apoptosis assays were performed using PI/RNase Staining Buffer and a PE Annexin V Apoptosis Detection Kit (BD Biosciences, San Jose, CA, USA), respectively. Staining cells were analyzed on a CytoFLEX flow cytometer (Beckman Coulter), and the data were further analyzed using FlowJo^TM^ v10.4 Software.

### RNA-seq profiling and analyses

Total RNAs were isolated from BACH2^**Con**^ and BACH2^**OE**^ Jurkat cells (*n* = 2/group) to generate RNA libraries, which were sequenced using Illumina HiSeq2500 (Gene Denove Biotechnology Co., Guangzhou, China). RNA-seq reads were aligned to the reference genomes (GRCh38) with Hisat2 (v2.2.1) [51] and quantified by StringTie (v2.1.2) [52] based on the reference human genome annotation file (Homo_sapiens.GRCh38.101.gtf). A R package DESeq2 [53] was used for DEGs examination. Genes with the adjusted *p* value < 0.05 and the absolute Log_2_(FC) ≥ 1 were considered DEGs, and volcano plot was generated using EnhancedVolcano R package (v1.18.0) (https://github.com/kevinblighe/EnhancedVolcano). The top 20 DEGs were used to generate heatmap. PCA was performed using the plotPCA function. To test the robustness of RNA-seq analysis, the supplementary file (GSE63602_DESeq_results.xlsx) from a GEO dataset (GSE63602) was downloaded for analysis of T-ALL patient samples vs. normal thymic T cells. Briefly, the baseMean values of genes were extracted and horizontally merged with the baseMean values of DEGs from BACH2^**OE**^ vs. BACH2^**Con**^ Jurkat cells, followed by Log10 transformation. R package pheatmap was used to generate heatmap with row scaling.

GO enrichment analysis was further performed using the clusterProfiler R package [54]. Weighted gene co-expression network analysis (WGCNA) was performed to analyze gene regulatory network using the R package WGCNA (v1.70) [55] to identify the gene co-expression modules with a high correlation with BACH2. PPI analysis of DEGs was performed using the STRING database (v11.5) (https://cn.string-db.org/) and the resulting network was visualized using the Cytoscape 3.9.1. The RNA-seq raw data can be found in the Sequence Read Archive (SRA). The BioProject accession number is PRJNA989395, and the SRA records will be accessible with the link of https://www.ncbi.nlm.nih.gov/sra/PRJNA989395 upon publication.

### CUT&Tag-seq analyses

CUT&Tag libraries were generated using Jurkat cells followed by sequencing on an Illumina NovaSeq 6000 sequencer (Biomarker Technologies, Beijing, China). Reads were aligned to the reference genomes (GRCh38) with Bowtie2 (v2.3.5.1) [56], and duplicated reads were removed using the MarkDuplicates tool of Picard (v2.21.6) (http://broadinstitute.github.io/picard/). Peaks calling was performed using MACS2 (v2.2.7.1) “callpeak” function [57] with a *p* value cutoff of 0.05. Annotation of peaks was performed using ChIPseeker [58] with default parameters. Integrative Genomics Viewer (IGV) v2.8.3 [59] was used to visualize the distribution of normalized sequence read density profiles. The overlapped genes between CUT&Tag-seq and RNA-seq (*n* = 129) were utilized for GO enrichment analysis using the clusterProfiler R package. HOMER [60] was used to perform Motif Enrichment Analysis with the findMorifsGenome.pl tool by default settings.

### Generation of truncated promoter constructs and luciferase activity assays

The truncated human *CD28* promoter and *CD40LG* promoter that contain putative BACH2-binding sites (Maf recognition elements, MARE) were amplified by PCR from genomic DNA. Two PCR products were inserted into pGL3-basic vectors (Promega, Madison, WI, USA) at KpnI and XhoI sites to make constructs of pGL3-CD28p and pGL3-CD40LGp, respectively. Two promoter constructs were further verified by sequencing. The pcDNA3.1-BACH2 plasmid was previously constructed [10]. Luciferase activity assay was performed and analyzed as previously described [10].

### Cell adhesion assays and cytokines analyses

Cell adhesion assay was performed as previously described [61]. To analyze cytokines, the manipulated T-ALL cells were plated onto the monolayer of HS-5 cells for 48 h in coculture medium (complete RPMI1640: complete DMEM = 1:1) under 5% CO_2_ at 37 °C. Coculture media were then collected to detect cytokines including IL-6 and IL-8 by ELISA assays (NeoBioscience, Shenzhen, China).

### Migration and chemotaxis assays

The manipulated T-ALL cells were pre-stained with PKH26 followed by the treatment with PBS, hCD28-mAb (R&D Systems, 0.5 μg/mL), rhCD40 (Abcam, 0.5 μg/mL) or both (hCD28-mAb+rhCD40) in RPMI1640 with no FBS. Cells were then added to transwell inserts with 8 μm pore size polycarbonate filters (Corning, Kennebunk, ME, USA) in a 24-well plate. Culture media containing the pre-established monolayer of HS-5 BMSCs or complete media with recombinant human CXCL12 (rhCXCL12, R&D Systems, 10 ng/mL) were placed in the lower wells. Chambers were incubated for 4 hours at 37 °C in 5% CO_2_, and cell migration was quantified by measuring PKH26 dye intensity in lower chambers.

### Tumor xenografts and treatment

Female NOD/SCID mice (6 weeks old, 18–20 g) were purchased from Charles River Laboratories (Beijing, China), and were housed in the barrier conditions at Institute of Medical Biology (IMB). All animal procedures were approved by the IMB Animal Care and Use Committee. Mice were pre-treated with an intraperitoneal (i.p.) injection of cyclophosphamide (CTX) at a dose of 100 mg/kg once daily for two consecutive days, followed by an intravenously (i.v.) injection with the manipulated Jurkat cells (2 × 10^6^ cells/mouse, *n* = 4/group) via tail vein. The xenograft mice were monitored twice a week, and humanely sacrificed by cervical dislocation when they presented leukemic phenotypes. Spleens and BMs were collected to analyze T-ALL infiltration via determining the percentage of GFP positive (GFP^+^) cells using flow cytometry.

In a sperate cohort, 10 days following transplantation of the BACH2^**OE**^ Jurkat cells, mice were then randomized into seven treatment groups: (1) saline (control, *n* = 5); (2) LD hCD28-mAb (20 μg/mouse, *n* = 4); (3) LD rhCD40 (20 μg/mouse, *n* = 4); (4) LD hCD28-mAb+rhCD40 (*n* = 4); (5) HD hCD28-mAb (100 μg/mouse, n = 4), 6) HD rhCD40 (100 μg/mouse, *n* = 4), and 7) HD hCD28-mAb+rhCD40 (*n* = 4). hCD28-mAb and rhCD40 used for in vivo experiments (Novoprotein, Shanghai, China) were dissolved and diluted in pyrogen-free sterile saline. Mice were administered by i.p. every three days for a total of 4 injections starting at day 11 and were humanely sacrificed 4 days following the final dose of treatment at day 24. Spleens and BMs were collected, and leukemic burden was evaluated by determining the percentage of GFP^+^ cells. During the experiment, the investigators were blinded to the group allocation of the animals. No statistical methods were used to predetermine the sample size, which was based on previous experimental observations, and no animals were excluded from the analysis.

### Statistical analysis

Data are presented as mean ± standard error of mean (SEM) or standard deviation (SD). Differences between two groups were determined by the two-tailed Student *t*-test; *p < 0.05 and **p < 0.01 were considered statistically significant. The variance was similar between the groups that were being statistically compared. Statistics were performed using Prism 9 (GraphPad, San Diego, USA). All experiments and assays were repeated at least three times and performed in duplicate or triplicate.

### Supplementary information

Supplementary data

Original Data

## Data Availability

The published article includes all data generated/analyzed for this study. Other relevant data are available from the authors upon request.
