# Supplementary material for: BACH2-mediated CD28 and CD40LG axes contribute to pathogenesis and progression of T-cell lymphoblastic leukemia
Source: Cell Death Dis. 2024 Jan 17;15(1):59. doi: 10.1038/s41419-024-06453-8 (PMC10794190; doi:10.1038/s41419-024-06453-8)
Supplement: Supplementary file 1 — Supplementary data [file 41419_2024_6453_MOESM1_ESM.pdf]

Supplementary Figure S1

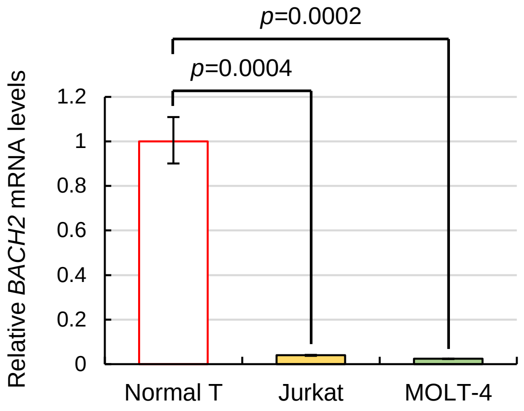

Supplementary Figure S1. Relative *BACH2* mRNA levels in human T-ALL cell lines (Jurkat and MOLT-4) vs. normal peripheral CD3^{+} T cells. Each value was normalized to *ACTB*, and data are shown as the mean ± SD.

Supplementary Figure S2

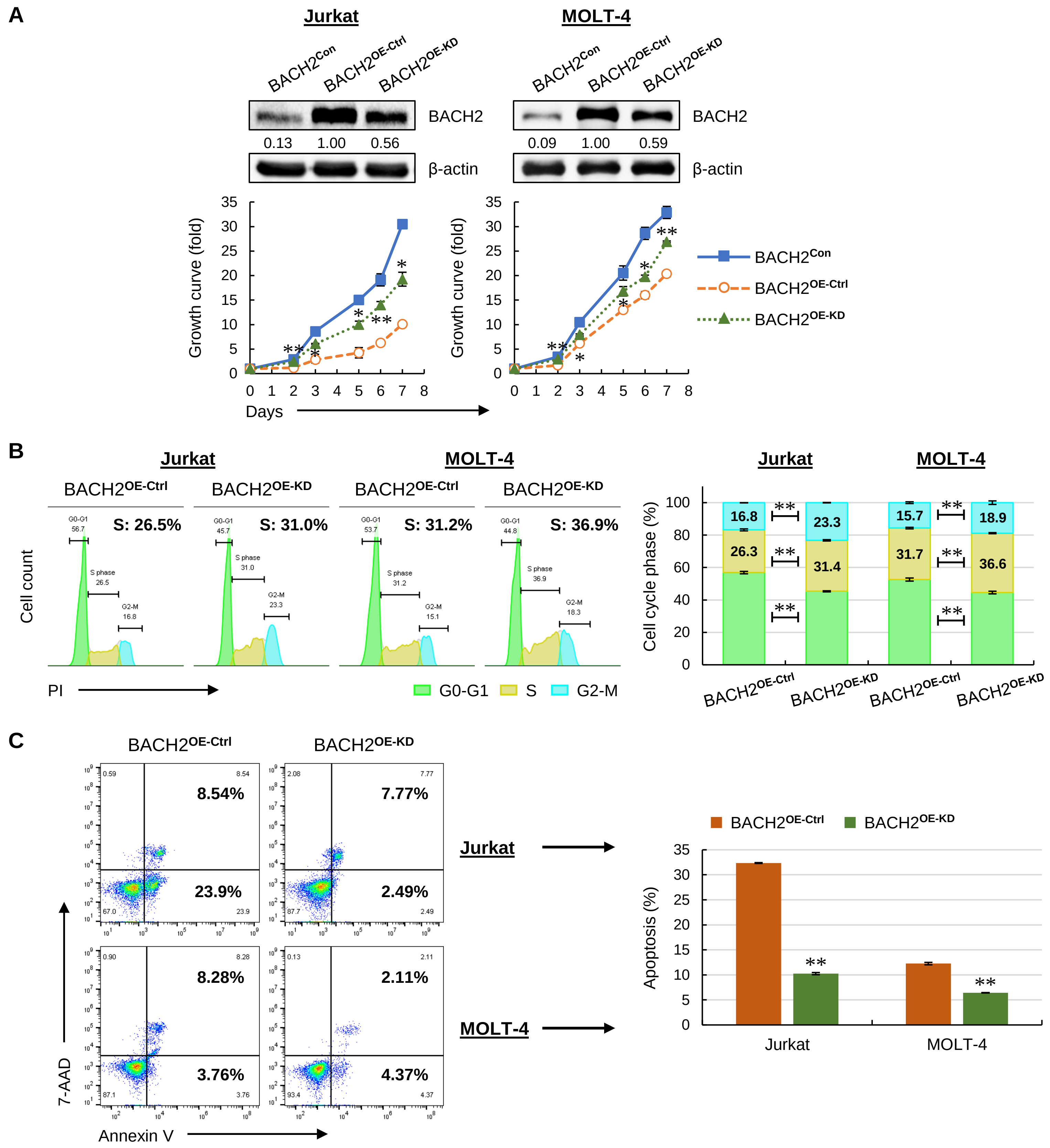

Supplementary Figure S2. (A) The knockdown efficiency of BACH2 (BACH2$^{OE\text{-}KD}$) in manipulated Jurkat (*left*) and MOLT-4 (*right*) cells were validated by immunoblots with a nonsilencing shRNA plasmid (BACH2$^{OE\text{-}Ctrl}$) as a negative control. β-actin was used as a loading control. Viable cells were counted in manipulated T-ALL cells (*lower*). **(B)** Representative cell-cycle distribution of manipulated Jurkat and MOLT-4 cells (*left*). The % population of cells in each phase is shown as the mean ± SD from three independent experiments (*right*). **(C)** Representative cell apoptosis in manipulated Jurkat and MOLT-4 cells staining with Annexin V/7-AAD (*left*). The % population of apoptotic cells in each group is shown as the mean ± SD from three independent experiments (*right*). **p*<0.05; ***p*<0.01 (vs. control group).

Supplementary Figure S3

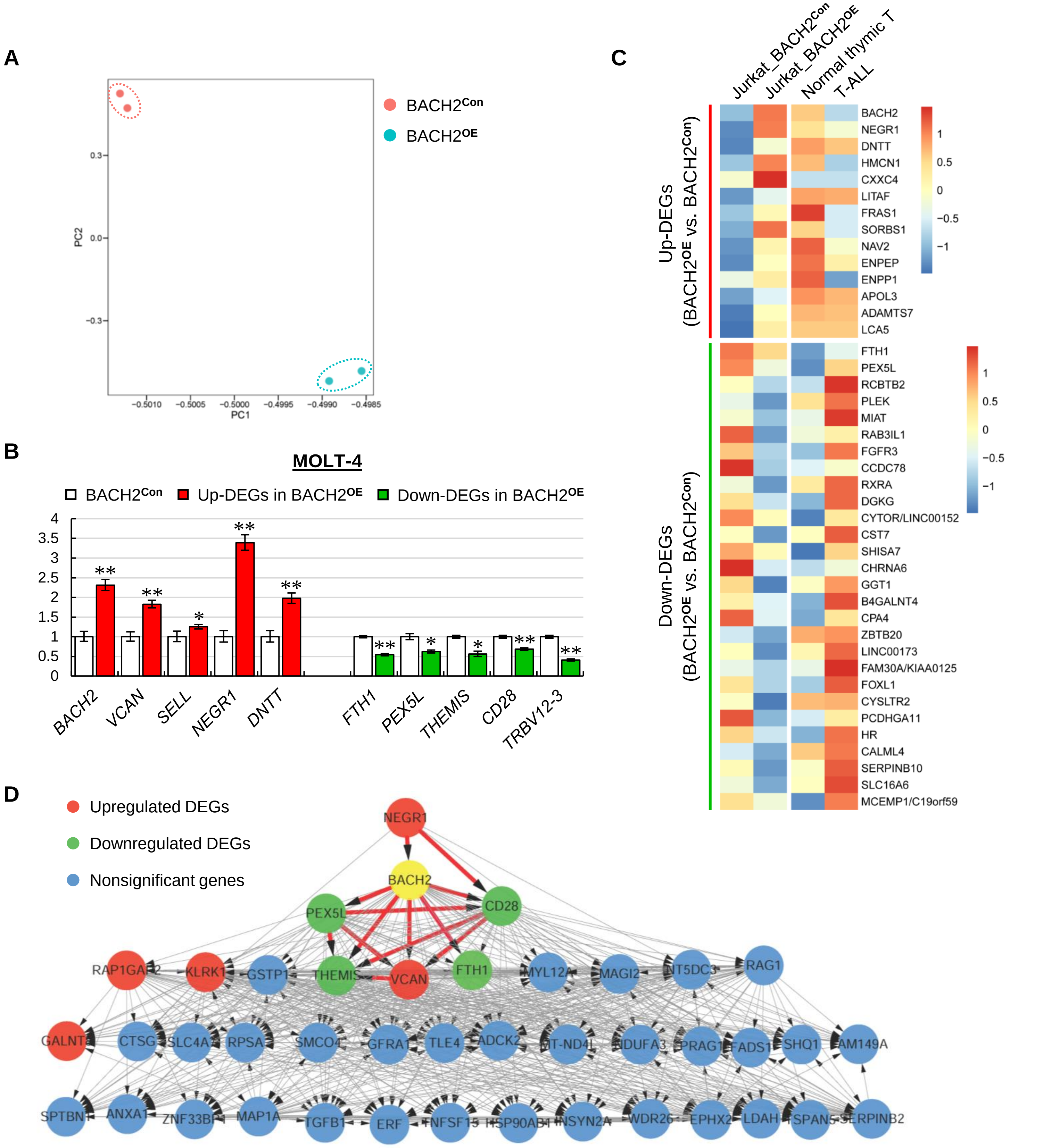

Supplementary Figure S3. (A) PCA of BACH2^{OE} Jurkat cells vs. control cells. **(B)** Relative mRNA levels of the top 5 Up- and Down-DEGs in manipulated MOLT-4 cells. Each value was normalized to *ACTB* and is presented as the mean ± SD from three independent experiments. **p*<0.05; ***p*<0.01 (vs. control group). **(C)** Heatmap of DEGs from BACH2^{OE} vs. BACH2^{Con} Jurkat cells as well as from T-ALL patient samples vs. normal thymic T cells (GSE63602). The level of gene expression is indicated by color intensity, with red representing high expression and blue representing low expression. **(D)** Hub genes in BACH2-involving gene regulatory network using weighted gene co-expression network analysis. The black arrows pointed to the downstream target genes and the lines indicated the gene-gene interactions.

Supplementary Figure S4

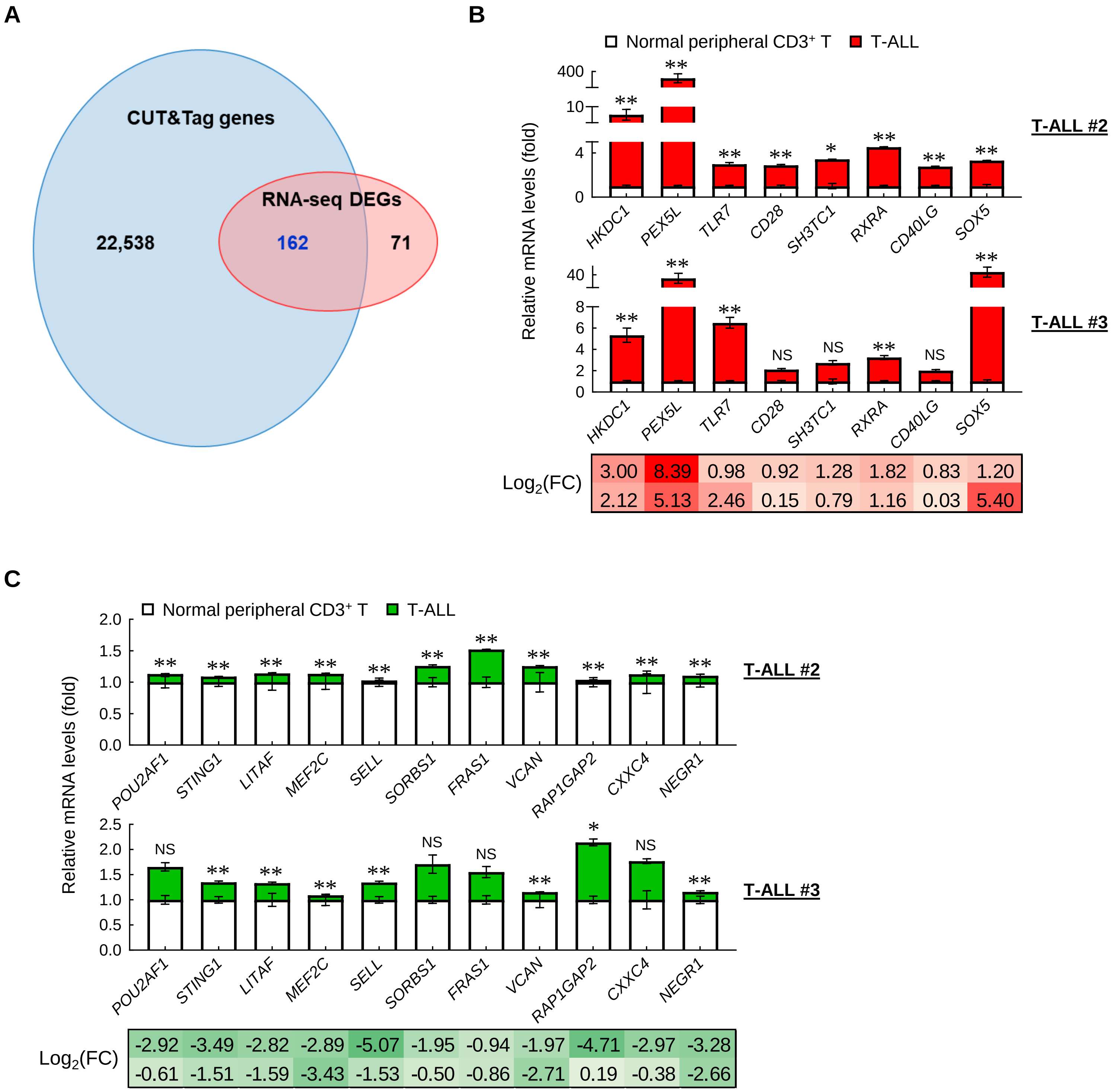

Supplementary Figure S4. (A) Venn diagram showing the overlap of annotated peaks identified from CUT&Tag-seq for BACH2 and the DEGs from RNA-seq. RNA-seq identified Down-DEGs **(B)** and Up-DEGs **(C)** were validated in two T-ALL patient samples (#2 and #3) vs. normal peripheral $CD3^+$ T cells. Each value was normalized to *ACTB* and is presented as the mean ± SD from three independent experiments. NS, not significant; **p*<0.05; ***p*<0.01 (vs. control group). $Log_2(FC)$ of each gene in T-ALL samples relative to the control cells is indicated under the bar chart, with the upper row representing T-ALL #2 and the lower row representing T-ALL #3.

Supplementary Figure S5

A

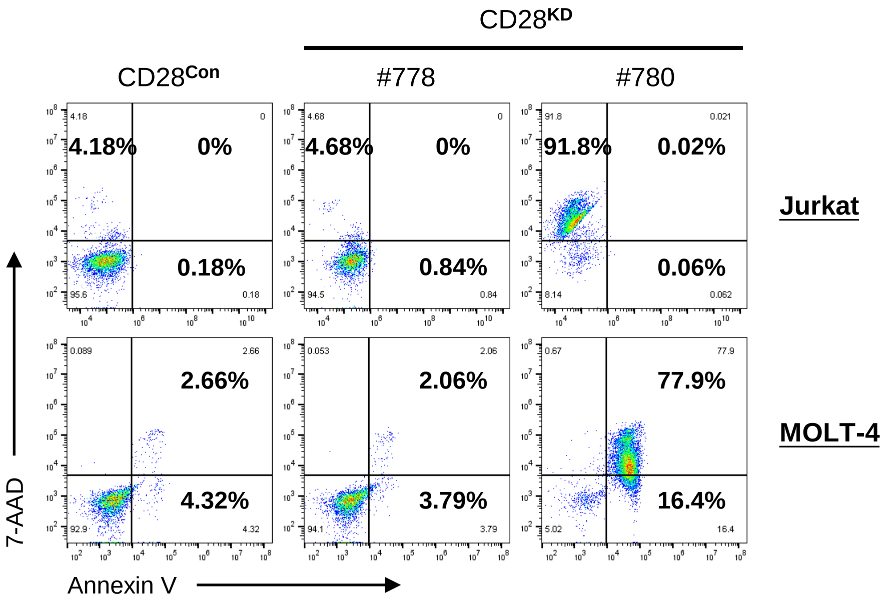

B

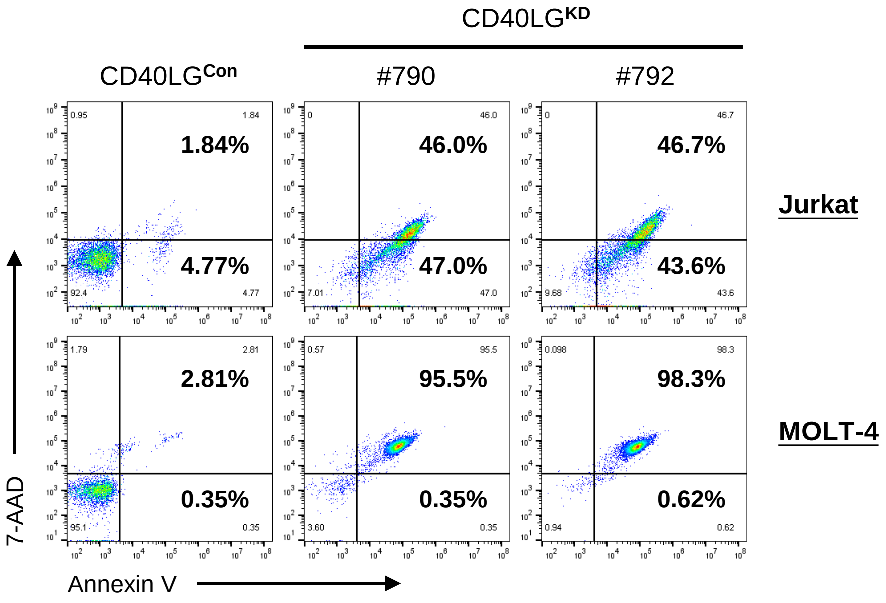

Supplementary Figure S5. Representative cell apoptosis and necrosis in manipulated Jurkat and MOLT-4 cells for CD28 **(A)** and CD40LG **(B)**.

Supplementary Table S1

Gene	Forward primer (5'-3')	Reverse primer (5'-3')
TRBV12-3	TTTGCTCATGCTCACAGAGGG	CAGGAGTCCATGGCAGAGTG
HKDC1	TGAGCCGTCTGACCAAAGC	TAGGGGTCGTCATAGGCACA
PEX5L	CCATCTCGGCTAGTGAGAAGG	TCTCCGCCTGGGTTATCCC
TLR7	CACATACCAGACATCTCCCCA	CCCAGTGGAATAGGTACACAGTT
CD28	CTATTTCCCGGACCTTCTAAGCC	GCGGGGAGTCATGTTCATGTA
SH3TC1	CTGCCCAAACTTCCCACTACC	CACGGACTCCGGCAATAGC
RXRA	GACGGAGCTTGTGTCCAAGAT	AGTCAGGGTTAAAGAGGACGAT
CD40LG	GAGCAACAACTTGGTAACCCT	GGCTGGCTATAAATGGAGCTTG
THEMIS	TCATAAAGCAGGGTGAGCAAAT	CGTTCATCTTCACACTCGTAGAA
SOX5	CAGCCAGAGTTAGCACAATAGG	CTGTTGTTCCCGTCGGAGTT
DNTT	GGGTCCAAGGTGAAGGGTATC	TTCAGCCCCACTCCAAATACA
POU2AF1	TGGACACCTTACACCGAGTAT	CGGAGAGGCATAGGTCAACAC
STING1	AGCATTACAACAACCTGCTACG	GTTGGGGTCAGCCATACTCAG
LITAF	ATGTCGGTTCCAGGACCTTAC	TACGAAGGAGGATTCATGCCC
MEF2C	CTGGTGTAACACATCGACCTC	GATTGCCATACCCGTTCCCT
EML1	TTCGTGGACGCCCTGTTAC	TGTACCCATAGACCCATTCCAG
SELL	ACCCAGAGGGACTTATGGAAC	GCAGAATCTTCTAGCCCTTTGC
SORBS1	ATTCCCAAGCCTTTCCATCAG	TTTTGCTGTTCTCGATTGTGTTG
FRAS1	CTAGCGTTGGCGGAATTTGC	GCATTGGTTGGCAGCTATTTGA
VCAN	GTAACCCATGCGCTACATAAAGT	GGCAAAGTAGGCATCGTTGAAA
RAP1GAP2	CCTGTCCGTCAAGTGCGAG	TACCGTCTTCAGTTTGGACCT
CXXC4	CCCGCAGAATCATTCCTCCTC	CCACAGTTGATGAGCCTCTTG
NEGR1	CAGACTCAACATACACCCAGAAC	AAACAAGTAAGAGTGACGTTGGT
