# Supplementary figures and images for: BACH2-mediated CD28 and CD40LG axes contribute to pathogenesis and progression of T-cell lymphoblastic leukemia
Source: Cell Death Dis. 2024 Jan 17;15(1):59. doi: 10.1038/s41419-024-06453-8 (PMC10794190; doi:10.1038/s41419-024-06453-8)

Figure 1

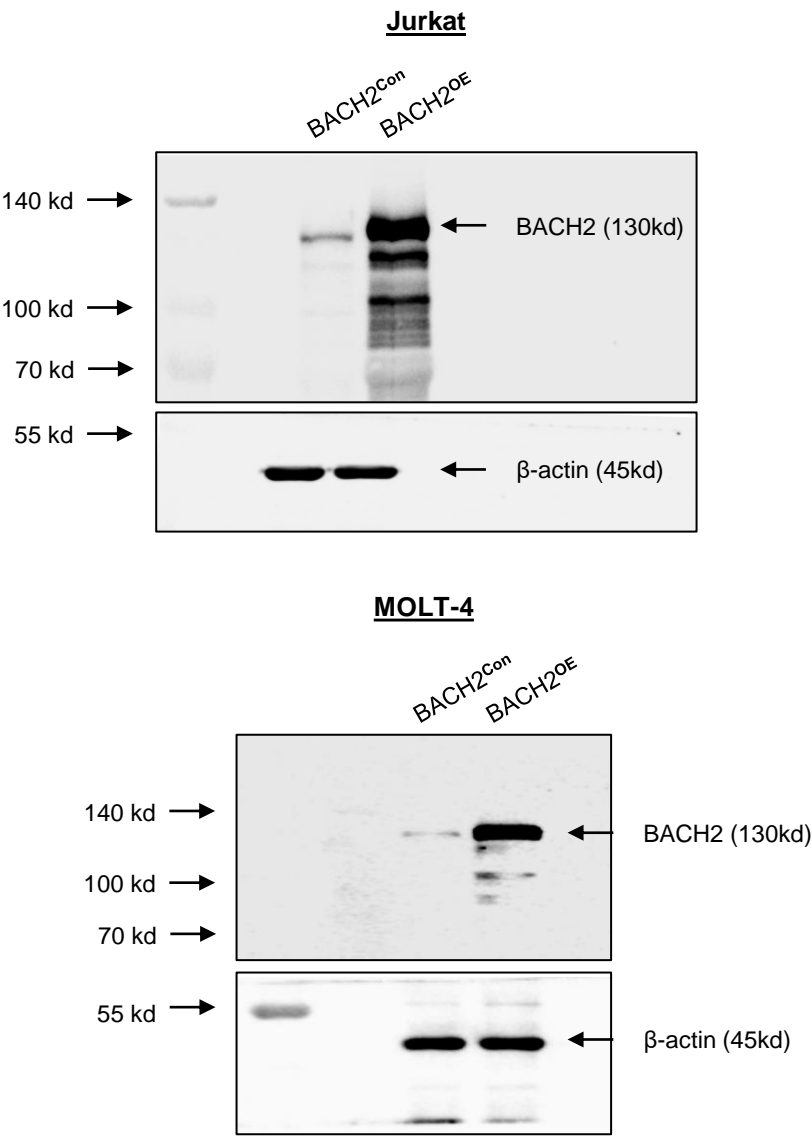

Figure S2

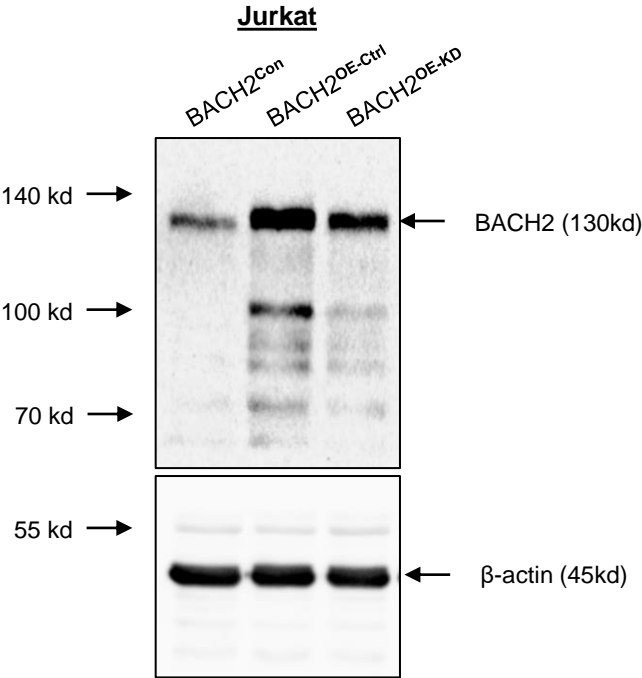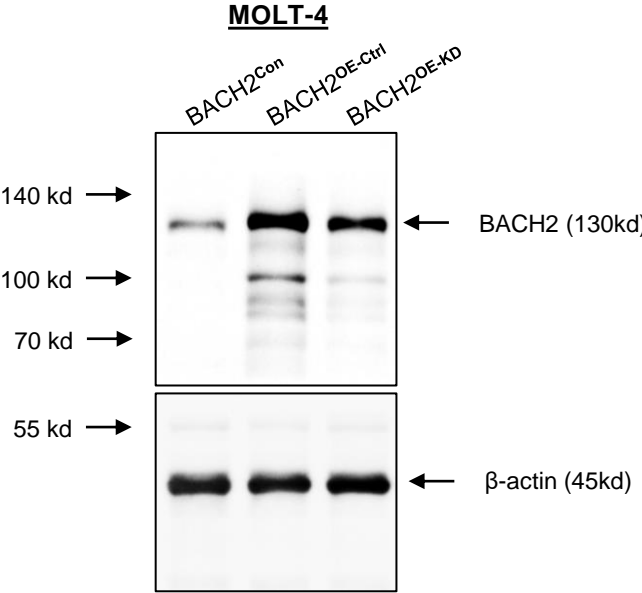

Supplement: Supplementary file 2 — Original Data [file 41419_2024_6453_MOESM2_ESM.pdf]
